# Quality of life of family carers of persons with young-onset dementia: A Nordic two-year observational multicenter study

**DOI:** 10.1371/journal.pone.0219859

**Published:** 2019-07-19

**Authors:** Lara Hvidsten, Knut Engedal, Geir Selbæk, Torgeir Bruun Wyller, Jūratė Šaltytė Benth, Hege Kersten

**Affiliations:** 1 Norwegian National Advisory Unit on Ageing and Health, Vestfold Hospital Trust, Tønsberg, Norway; 2 Division for Mental Health and Addiction, Vestfold Hospital Trust, Tønsberg, Norway; 3 Vestfold Hospital Trust, Tønsberg, Norway; 4 Oslo University Hospital, Department of Geriatric Medicine, Oslo, Norway; 5 Faculty of Medicine, University of Oslo, Oslo, Norway; 6 Institute of Clinical Medicine, Campus Ahus, University of Oslo, Oslo, Norway; 7 Health Services Research Unit, Akershus University Hospital, Lørenskog, Norway; 8 Pharmaceutical Bioscience, School of Pharmacy, University of Oslo, Oslo, Norway; 9 Department of Research and Development, Telemark Hospital, Skien, Norway; Nathan S Kline Institute, UNITED STATES

## Abstract

**Objectives:**

To identify factors associated with QOL in carers of persons with young-onset Alzheimer’s (AD) and frontotemporal dementia (FTD) and explore development in QOL over a two-year period.

**Methods:**

Eighty-eight family carers of community-dwelling people with young-onset AD (n = 50) and FTD (n = 38) recruited from Nordic memory clinics. Carer QOL was assessed using the Quality of Life–Alzheimer’s Disease questionnaire. Carer burden was assessed by the Relatives’ Stress scale and depressive symptoms by the Montgomery-Åsberg Depression Rating Scale. Factors associated with QOL in YOD and development in QOL over time were explored with growth mixture model trajectories and mixed model analyses.

**Results:**

We identified two carer groups of persons with YOD following trajectories with better (n = 53) versus poorer (n = 30) QOL. Carers who reported more burden at baseline had greater odds of belonging to the poorer QOL group (OR 1.1 (1.0–1.2), p = 0.004). Analyses of the development in QOL showed a significant decline in QOL–AD scores among the AD-carers from baseline to two-year follow-up (p = 0.044), while the score remained stable among the FTD-carers. The FTD-carer group had significantly higher mean QOL–AD scores at one- and two-year follow-up (p = 0.022 and 0.045, respectively). However, the difference between the two groups regarding time trend was non-significant. Poorer QOL was associated with increased carer burden (p = 0.01), more depressive symptoms (p = 0.024), and being male carer (p = 0.038).

**Conclusion:**

Higher care burden, more depressive symptoms, and being a male carer was associated with poorer QOL in family carers for persons with YOD. Carers of persons with AD may experience greater challenges in preserving QOL compared to carers of persons with FTD.

## Introduction

The symptom presentation in dementia is primarily determined by the affected brain areas, which causes the characteristic symptom profiles in two common dementia subtypes, Alzheimer’s (AD) and frontotemporal dementia (FTD). AD is in most cases initially associated with memory impairment whereas personality and behavioral changes, or language problems are prominent early features in FTD. Different symptom profiles are likely to have different impact on family carers and possibly also affect quality of life (QOL) [1–4]. As both AD and FTD lead to progressive impairment of various brain functions family carers find themselves dedicating increasingly more time and effort to informal care at the expense of other tasks. Dementia has been said to affect the family even more than the individual receiving the diagnosis, as a condition with an “invisible second patient” [5, 6]. The impact of caring is accentuated in young-onset dementia (YOD) as the dementia symptoms start before the age of 65, during the most active and productive years of life. The repercussions to the individual and their families are greater [7] as care responsibilities may be combined with a working career, childcare, social obligations, and hobbies and interests. Balancing these competing tasks whilst maintaining good physical and mental health, and QOL, can be a challenge [8–11], and failure to do so may result in a sense of entrapment in the caring role.

The prevalence of depression in family carers in YOD is high [12, 13] with high levels of burden [5, 14, 15] and poorer health-related QOL compared to the general population [16]. Negative health outcomes are partly mediated by physiologic immunologic and neuroendocrine responses to the prolonged strain of caring [5, 17, 18]. An earlier US study demonstrated a 63% increase in all-cause four-year mortality in a large cohort of spouses (mean age 80 years, non-dementia specific carers) who reported mental or emotional strain compared to non-carers, adjusted for e.g. age, sex, education, and physical health status [19]. On the other hand, these mortality rates have since been disputed in population-based studies [20]. Also, several other studies have reported that whilst caring is often a stressful experience, there is a significant proportion of carers who do not experience strain (i.e. 44% in the US study), or report mixed or even positive experiences [20–24]. These positive aspects are offered less attention, and as stated in a 1997-review encompassing gains in caring, “the lack of attention to the positive dimension of caregiving seriously skews perceptions of the caregiving experience” [21].

Several studies have also found QOL within dyads to be inter-linked [25–28]. One study showed that people with dementia perceived better QOL when their families reported less stress related to care [29], indicating that healthy carers provide better quality care.

In previous research, depression has been identified as the strongest and most consistent factor associated with poorer QOL in carers of persons with late-onset dementia [30]. Recurrent depression has been associated with more rapid decline in health compared to non-depressed carers [31], and depressed carers with compromised QOL are more likely to resort to institutionalization [32, 33]. As few studies have specifically assessed factors associated with QOL of family carers in YOD [4, 34], including QOL as an outcome measure has been requested [4, 9, 34]. Moreover, there is a need to explore differences between the diagnostic subtypes with regard to impact on QOL [2]. Thus, the aims of the present study were to explore the development of QOL of family carers of persons with young-onset Alzheimer’s and frontotemporal dementia over a two-year period, to identify potential groups of carers following different trajectories of QOL, and assess covariates associated with time trend within the two diagnostic groups.

## Materials and methods

### Participants

This was a two-year prospective Nordic cohort study of family carers of home-dwelling persons with young-onset AD (n = 50) and FTD (n = 38). The term “family carer” was used in the extended meaning of the term, including any significant other providing unpaid informal help. The family carers and persons with dementia were recruited in dyads from nine memory clinics in Norway, Denmark and Iceland from February 2014 to July 2015 [35].

All the recruiting centres were specialized hospital clinics, either on a secondary and/or tertiary level. In the Nordic countries, apart from Iceland, basic dementia work-up is conducted by the primary health care services according to national guidelines. More complex dementia diagnostics, such as in persons suspected of having YOD, is a designated task for the specialized health care services. The organizational structure of each memory clinic (within Neurology, Geriatrics or Psychiatry) may vary with location, also within each country. However, the diagnostic procedures in the Nordic countries have been compared and found to be similar in the Nordic Network in Dementia Diagnostics [36].

The Nordic project nurses were trained co-workers recruited locally at each clinic and designated for the task throughout the study period. As they were already familiar with most of the assessments used in the study as part of the regular dementia work-up, orientation meetings were held concerning the study-specific assessments that were not a part of the usual clinical work-up. In Norway, the same ambulatory team of one physician (author) and two project nurses conducted all the assessments.

The QOL of the persons with dementia was described in a previous study [37]. The only inclusion criteria for the family carers were face-to-face contact with the person with dementia at least once weekly and written informed consent to participation. The assessments were made as part of semi-structured interviews by a physician and project nurse at baseline, and at one- and two-year follow-up, Table 1.

**Table 1:** The assessments of family carers and persons with dementia.

VARIABLE	INSTRUMENT	INFORMATION SOURCE		
*Family carer*		Baseline	One-year	Two-years
Depression	MADRS	FC	FC	FC
	GDS	FC	FC	FC
Burden	RSS	FC	FC	FC
QOL	QOL-AD	FC	FC	FC
*Person with dementia*		Baseline	One-year	Two-years
Dementia severity	CDR	R	R	R
Cognition	MMSE	p	p	p
Depression	CSDD	FC	FC	FC
Awareness	Reed scale	P/FC/R	P/FC/R	P/FC/R
ADL	I-ADL	FC	FC	FC
	PSMS	FC	FC	FC
QOL	QOL-AD	FC	FC	FC

P = Person with dementia, FC = Family carer, R = Researcher. MADRS = Montgomery-Åsberg Depression Rating Scale, GDS = Geriatric Depression Scale, RSS: Relative’s Stress Scale, QOL-AD = Quality of life—Alzheimer’s Disease, CDR = Clinical Dementia Rating scale, MMSE = Mini Mental State Examination, CSDD = Cornell Scale for Depression in Dementia, I-ADL = Instrumental Activities of Daily Living, PSMS = Physical Self Maintenance Scale.

The interviews were held in parallel sessions with the persons with dementia and their family carers, either at the memory clinic or at home. The scales and questionnaries used in this study were either designed for self-assessment (e.g. Relative’s Stress Scale) or clinical interviews (e.g. Montgomery-Åsberg Depression Rating Scale). The most appropriate way of collecting the data could vary, but primarily as an interview rather than a survey. The reason for choosing this approach was to preserve the participants needs of conveying their individual stories, not just providing information to the study. The questionnaires were used as a structure to make sure all study items were covered appropriately.

### Characteristics of the family carers

The manual of the Norwegian register for persons with cognitive symptoms (NorCog) was used to assess sociodemographic and clinical variables of the family carers. This is a diagnostic manual comprising semi-structured interviews, cognitive tests and informant questionnaires, implemented as a standardized first-visit assessment routine in collaborating memory clinics in Norway [37, 38]. It also includes the Relatives’ Stress Scale (RSS) [39] as a screening tool for carer burden. This questionnaire consists of 15 statements scored on a five-point scale from 0 = not at all to 4 = considerably, with a total score ranging from zero to 60, higher scores indicating greater burden [40]. The Montgomery-Åsberg Depression Rating Scale (MADRS) [41] measured depressive symptoms, consisting of ten items rated from zero to 6 with a total score ranging from zero to 60; a cut-off score of seven or higher indicating depression [42, 43]. The Resource Utilization in Dementia (RUD) Lite [44] was used for assessing the hours of informal assistance provided by the family carers for persons with dementia living at home.

The Quality of Life—Alzheimer’s Disease (QOL–AD) questionnaire was used to assess quality of life [45]. This questionnaire consists of 13 items; physical health, energy, mood, living situation, memory, family, marriage, friends, self as a whole, ability to do chores around the house, ability to do things for fun, money, and life as a whole. The items were rated on a 4-point scale from poor to excellent, with a total score ranging from 13 to 52, higher score indicating better quality of life. According to Conde-Sala et al., QOL–AD scores above 37 can be regarded as good QOL and QOL–AD scores below 33 as poor QOL [46].

### Characteristics of the persons with YOD

Sociodemographic, clinical and functional characteristics of the persons with dementia were also assessed using the NorCog diagnostic manual. Diagnosis had been established as part of the diagnostic work-up in the memory clinics prior to study inclusion, according to the International Classification of Diseases-10th revision criteria for Alzheimer’s dementia, and the Neary et al. or the International consensus criteria for behavioral variant of frontotemporal dementia [47, 48], or the Mesulam criteria for the language variant [49]. The Clinical Dementia Rating scale sum-of-boxes score was used to assess dementia severity [50], the Cornell Scale for Depression in Dementia was used to measure depression [51], and disease awareness was classified into four categories according to the Reed anosognosia scale [52].

The QOL of the persons with dementia was assessed using the proxy version of the QOL–AD [53]. In the present study, the families were instructed to apply the “proxy-patient perspective” [54]; i.e. report how the persons with dementia would rate their own QOL.

### Statistical analyses

Distribution of variables was examined using histograms. Categorical variables were described by their frequencies and percentages, continuous variables by their means and standard deviations (SD). Comparisons of carers of persons with AD or FTD were assessed by χ^2^-test or Independent Samples t-test as appropriate.

Growth mixture model was estimated to identify possible groups of family carers following distinct trajectories of QOL–AD throughout the two-year study period. The Akaike Information Criterion (AIC) was used to determine the optimal number of groups. Average within-group probabilities were expected to be larger than 0.7, with non-overlapping 95% confidence intervals (CI) for trajectories. The identified groups were described by bivariate and multiple generalized linear models with group membership as dependent variable and selected baseline covariates as explanatory variables. Random effects for center were included. Based on clinical considerations, previous research and correlations among covariates, a reduction of covariates was made from an initial list of characteristics (e.g. living situation, met/unmet needs, Neuropsychiatric Inventory Questionnaire severity score, Mini Mental State Examination, number of children age < 20 years).

Linear mixed model was estimated to explore overall time trend in QOL–AD throughout the study period. The model included random intercepts for family carers nested within center. Fixed effects for the same selected covariates as in the above analysis were included. Bivariate and multiple models were estimated. Interactions between each covariate and diagnosis (AD or FTD) were entered into the multiple model to assess differences between the diagnostic groups. Significant interaction implies that association between a certain covariate and QOL–AD differs in the two diagnostic groups.

Both multiple models were reduced by applying the AIC, where the smaller value means better model. Only interactions with p < 0.20 in the multiple models were retained.

The analyses were performed using the IBM SPSS v 24 and STATA v 14. All testes were two-sided and results with p-values below 0.05 were considered statistically significant.

### Ethical considerations

The project was approved by the Norwegian Regional Committees for Medical and Health Research Ethics. The research was performed in accordance with the World Medical Association’s Declaration of Helsinki—Ethical Principles for Medical Research Involving Human Subjects. Participation required informed written consent from the family carers and the persons with dementia.

## Results

**Fig 1:**
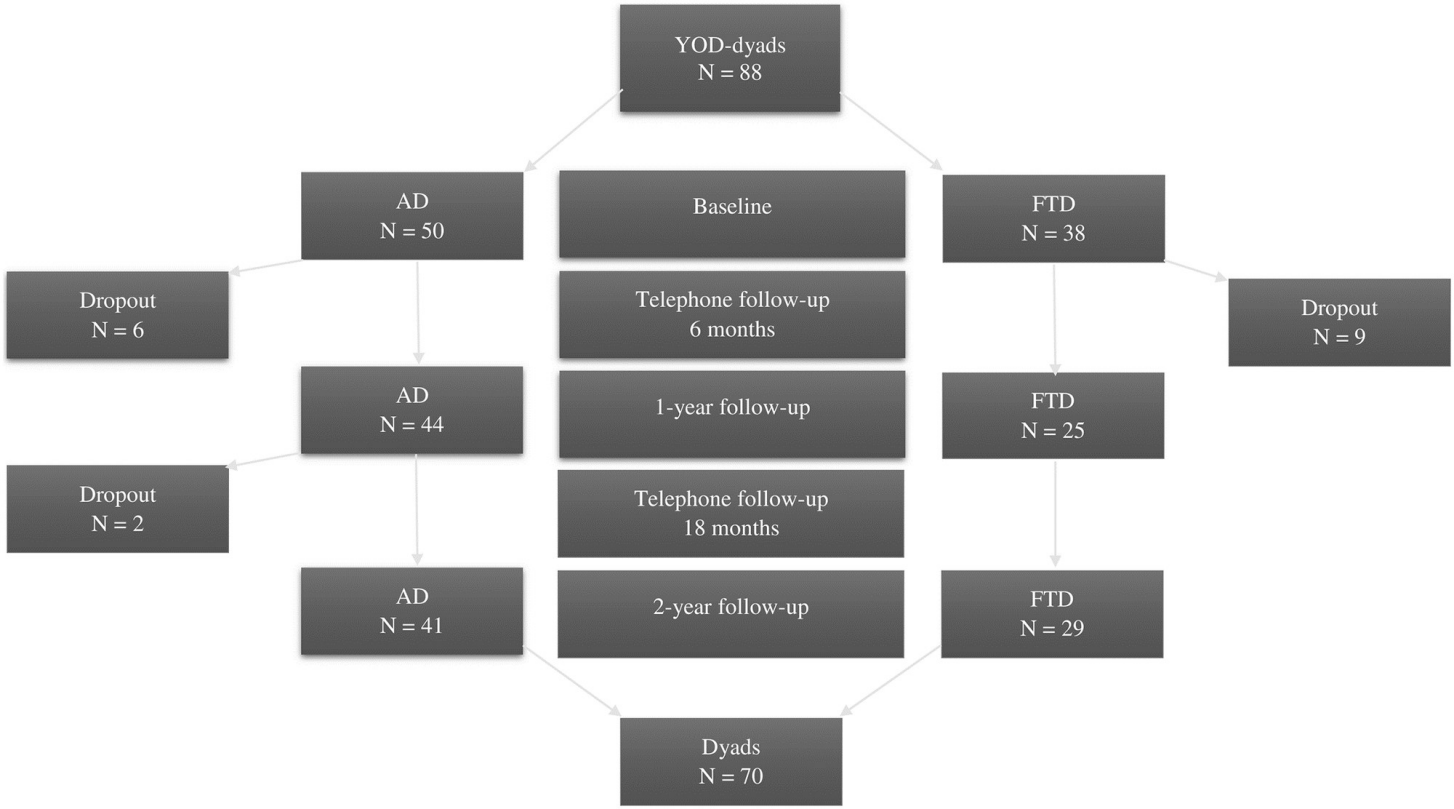
Flow chart of the family carers in young-onset dementia, assessment time points and dropouts.

Of the included 88 family carers 70 (80%) completed the two-year follow-up, Fig 1.

Dropout was mainly due to factors related to the persons with dementia or the total strain on the families. Additionally, in five cases new family carers were introduced at follow-up (these data were omitted from the longitudinal analyses). In two cases carers completed follow-up after the person with dementia had deceased. There were no significant baseline differences in age, sex, diagnosis, dementia severity, or scores on the Relatives’ Stress Scale, Montgomery-Åsberg Depression Rating Scale or QOL–AD between carers who completed the follow-up and those who dropped out.

Descriptive data from baseline, and at one- and two-year follow-up for the family carers and the persons with dementia are shown in Table 2.

**Table 2:** Characteristics of the family members and persons with young-onset dementia at baseline, and one- and two-year follow-up.

Characteristics	Baseline	One-year	Two-year
*Family member*			
Number, n	88	68	64
Age, mean (sd)	57 (11.7)	-	-
Male, n (%)	36 (41)	28 (41)	25 (39)
Relationship			
Spousal	61 (70)	50 (74)	48 (74)
Other	26 ((30)	18 (26)	16 (25)
Montgomery-Åsberg Depression Rating Scale	7.0 (7.7)	7.2 (6.6)	7.6 (5.8)
Geriatric Depression Scale	6.7 (5.8)	7.6 (6.8)	7.1 (6.7)
Relatives’ Stress Scale	18.7 (12.4)	21.6 (12.1)	18.9 (12.2)
ADL-assistance, hrs per day	3.2 (4.8)	3.4 (5.0)	4.4 (6.1)
QOL–AD	38.4 (6.5)	37.2 (7.0)	36.2 (7.3)
*Person with dementia*			
Number, n	88	68	64
Dementia diagnosis, n			
Alzheimer’s	50	49	40
Frontotemporal	38	37	24
Age	63.0 (4.8)	-	-
Male, n (%)	48 (55)	34 (50)	33 (48)
Clinical Dementia Rating^α^	4.9 (3.4)	6.8 (4.6)	9.1 (5.2)
Mini Mental Status Examination	21.6 (6.5)	20.4 (6.0)	17.7 (8.1)
Symptom duration, years	4.8 (2.7)	-	-
Cornell Scale for Depression in Dementia	7.0 (5.6)	7.9 (6.0)	8.0 (4.9)
Awareness, n (%)			
Intact	51 (60)	27 (53)	15 (33)
Impaired	34 (40)	24 (47)	31 (67)
Activities of Daily Living	21.3 (7.8)	26.0 (10.1)	25.3 (9.7)
QOL–AD	36.3 (6.6)	35.6 (5.2)	34.3 (6.6)

Mean, SD unless specified otherwise. QOL–AD = Quality of Life—Alzheimer’s Disease, ADL = Activities of Daily Living.

^α^ Clinical Dementia Rating sum of boxes score.

Mean carer age was 57 years (SD 11.7), ranging from 25 to 75 years. At baseline, 70% of the family carers were spouses, 18% were adult children and 12% sibling or friend. There were no significant baseline differences in sex distribution or QOL–AD scores between carers of persons with AD or FTD. At two-year follow-up, one third (34%) of the persons with dementia had become nursing home residents.

### The trajectories of QOL–AD

According to the growth mixture model for QOL–AD, two groups of family carers with distinct trajectories in QOL–AD were identified. The average probabilities were high in both groups (0.92 and 0.90) with non-overlapping 95% CI clearly indicating two distinct groups of carers. The larger group consisting of n = 53 (64%) carers had a mean QOL–AD score of 41.5 (SE = 0.8) at baseline and displayed a linear stable pattern (p = 0.415 for slope) in QOL–AD throughout follow-up, hereby referred to as the “better QOL” group. The lesser group consisting of n = 30 (36%) carers with mean QOL-AD of 33.7 (SE = 1.0) at baseline showed a significant linear decline (p = 0.002 for slope) in QOL–AD, hereby referred to as the “poorer QOL” group.

The descriptive characteristics of the two trajectory-groups are shown in Table 3.

**Table 3:** Descriptive statistics of the two trajectory-groups (poorer and better QOL groups).

Characteristics	Poorer QOL	Better QOL
Sex, person with dementia		
Male, N (%)	20 (67)	26 (49)
Female, N (%)	10 (33)	27 (51)
Diagnosis		
AD, N (%)	18 (60)	32 (60)
FTD, N (%)	12 (40)	21 (40)
Clinical Dementia Rating		
Mean (SD)	5.9 (3.2)	4.2 (3.4)
Cornell Scale for Depression in Dementia		
Mean (SD)	9.8 (6.8)	5.4 (4.0)
Awareness		
Intact, N (%)	12 (41)	38 (75)
Impaired, N (%)	17 (59)	13 (25)
QOL, person with dementia		
Mean (SD)	34.4 (7.2)	37.8 (5.9)
Sex, family member		
Male, N (%)	11 (37)	23 (43)
Female, N (%)	19 (63)	30 (64)
Age, family member		
Mean (SD)	57.5 (11.1)	57.2 (11.8)
Relationship		
Spouse, N (%)	21 (70)	37 (70)
Adult child, N (%)	4 (13)	11 (201)
Other, N (%)	5 (17)	5 (9)
Montgomery-Åsberg Depression rating Scale, family member		
Mean (SD)	11.5 (9.6)	4.6 (4.9)
Relatives’ Stress Scale		
Mean (SD)	26.8 (10.8)	13.7 (10.4)

Table 4 presents the results of logistic regression models assessing potential predictors for QOL-group belonging.

**Table 4:** Variables associated with belonging to the “poorer QOL”, N = 97 (adjusted for people nested within centers).

Characteristics	Bivariate models		Multiple model		Multiple model, AIC-reduced	
	OR (95% CI)	p-value	OR (95% CI)	p-value	OR (95% CI)	p-value
Diagnosis						
Frontotemporal dementia	1.6 (0.5; 5.7)	0.457	0.2 (0.0; 1.8)	0.159		
Sex, person with dementia	0.43 (0.2; 1.2)	0.112	0.14 (0.0; 1.3)	0.083		
Clinical Dementia Rating	1.2 (1.0; 1.4)	0.035	0.8 (0.6; 1.1)	0.144		
Cornell Scale for Depression in Dementia	1.2 (1.1; 1.4)	0.001	1.1 (0.9; 1.3)	0.335		
Awareness						
Impaired	8.7 (2.2; 35.6)	0.003	4.6 (0.6; 33.9)	0.141	4.6 (0.8; 25.6)	0.078
QOL–AD, person with dementia	0.9 (0.8; 1.0)	0.009	0.9 (0.8; 1.1)	0.189	0.9 (0.8; 1.0)	0.051
Sex, family carer						
Female	1.3 (0.5; 3.6)	0.600	0.3 (0.0; 2.8)	0.313		
Age, participant	1.0 (1.0; 1.0)	0.899	1.0 (1.0; 1.1)	0.373		
Relationship						
Other	1.0 (0.4; 2.9)	0.977	2.4 (0.2; 24.4)	0.467		
Montgomery-Asberg Depression Rating Scale	1.2 (1.1; 1.3)	0.001	1.0 (0.9; 1.2)	0.539		
Relatives’ Stress Scale	1.1 (1.1; 1.2)	<0.001	1.1 (1.0; 1.2)	0.015	1.1 (1.0; 1.2)	0.004

Reference categories set to “better QOL” group, Alzheimer’s dementia, intact awareness, male and spousal relationship.

Burden, awareness, and the QOL–AD scores of the persons with dementia were retained in the multiple AIC-reduced model, but only higher burden measured by the Relatives’ Stress Scale was significantly associated with belonging to the poorer QOL-group (OR 1.1 (1.0–1.2), p = 0.004).

### Two-year development in QOL and associated factors among all carers assessed simultaneously

Table 5 shows the variables associated with QOL–AD time trend for all family carers combined.

**Table 5:** Variables associated with QOL–AD time trend (adjusted for people nested within centers).

Characteristics	Bivariate models		Multiple model		Multiple model, AIC-reduced	
	Regr.coeff. (SE)	P-value	Regr.coeff. (SE)	P-value	Regr.coeff. (SE)	P-value
Time						
One year	-1.6 (1.0)	0.98	-1.4 (0.9)	0.135	-1.6 (0.9)	0.100
Two years	-1.9 (1.0)	0.57	-1.8 (1.0)	0.068	-2.0 (1.0)	0.044
Diagnosis						
FTD	-0.9 (1.5)	0.550	13.1 (9.1)	0.154	2.9 (1.7)	0.091
Time x D						
One year	1.3 (1.6)	0.397	1.2 (1.6)	0.442	1.4 (1.6)	0.361
Two years	0.8 (1.6)	0.642	0.7 (1.6)	0.689	1.0 (1.6)	0.554
Sex, person with dementia						
Female	3.3 (1.7)	0.053	2.6 (2.2)	0.227	4.5 (1.7)	0.012
Clinical Dementia Rating	-0.4 (0.2)	0.073	0.2 (0.2)	0.315		
Cornell Scale for Depression in Dementia	-0.3 (0.2)	0.056	-0.1 (0.2)	0.560	-0.2 (0.1)	0.115
Awareness						
Impaired	-3.1 (1.9)	0.103	-0.6 (1.2)	0.654	-0.2 (1.3)	0.899
QOL-AD, person with dementia	0.3 (0.2)	0.074	0.0 (0.1)	0.841		
Sex, family carer						
Female	-2.3 (1.7)	0.189	0.9 (2.1)	0.651	3.0 (1.4)	0.038
Age, family carer	0.0 (0.1)	0.986	-0.1 (0.1)	0.557		
Relationship						
Other	1.7 (1.9)	0.368	-0.2 (2.3)	0.942	-1.1 (1.3)	0.365
Montgomery-Åsberg Depression Rating Scale, family carer	-0.4 (0.1)	<0.001	-0.2 (0.1)	0.048	-0.2 (0.1)	0.024
Relative Stress Scale	-0.2 (0.1)	<0.001	-0.2 (0.1)	0.003	-0.2 (0.1)	0.013
Sex, person with dementia x D						
Female	-5.0 (2.8)	0.074	-2.4 (2.9)	0.418	-4.5 (2.4)	0.060
Clinical Dementia Rating x D	-0.2 (0.4)	0.602				
Cornell Scale for Depression in Dementia x D	-0.4 (0.2)	0.058	-0.3 (0.2)	0.197		
Awareness x D						
Impaired	-1.1 (2.8)	0.696				
QOL-AD, person with dementia x D	-0.0 (0.2)	0.848				
Sex, family member x D						
Female	5.6 (2.8)	0.044	1.6 (2.8)	0.567		
Age x D	-0.1 (0.1)	0.155	-0.1 (0.1)	0.267		
Relationship x D						
Adult child/others	-3.9 (2.9)	0.188	-4.0 (3.1)	0.199		
Montgomery-Åsberg Depression Rating Scale, family carer x D	-0.0 (0.2)	0.946				
Relative Stress Scale x D	-0.1 (0.1)	0.259				

QOL–AD = Quality of Life Alzheimer’s disease. AD = Alzheimer’s dementia; FTD = Frontotemporal dementia. Reference categories set to AD, male, intact awareness, and spousal relationship.

Even though in multiple AIC-reduced model there was a significant decline in QOL–AD scores in the AD-carers from baseline to two-year follow-up (p = 0.044) while the score remained stable in the FTD-carers, there was no significant difference between the two diagnostic groups regarding time trend (no significant interaction between time and diagnosis group). The FTD-carer group had however a significantly higher mean QOL–AD score at one- and two-year follow-up (p = 0.022 and p = 0.045, respectively). Interaction between sex of the person with dementia and diagnosis was also left in the multiple AIC-reduced model. Carers of persons with AD reported significantly higher QOL–AD scores when caring for women as compared to men (p = 0.012). However, there were no significant differences between the AD- and FTD-groups regarding time trend in QOL-AD when caring for women or men (p = 0.060). Furthermore, lower QOL–AD scores were significantly associated with higher levels of burden (p = 0.013) and depressive symptoms (p = 0.024) of the family carers. Higher QOL–AD scores were significantly associated with being female carer (p = 0.038). Multiple AIC-reduced model explained nearly 50% of between-carer variance in QOL-AD score.

## Discussion

This is one of few studies exploring the QOL of family carers of persons with YOD. The dyads were recuited in a Nordic multicentre collaboration. Nordic countries enjoy high standards of living, social benefits and well-developed, public health care systems based on equal social rights independent of economic status. Provision of comprehensive care on demand is mainly a statutory responsibility, and the health care services in the Nordic countries share basic similarities in organizational structures and diagnostic dementia work-up. Nordic countries are ranked among the top ten listed on the World Happiness Index [55].

Although two-thirds of the family carers reported QOL to be good throughout the two-year study period, overall QOL for all family carers declined from baseline to follow-up. The deterioration in QOL was explained by a significant decline in QOL in carers of persons with AD, while QOL in carers of persons with FTD was higher and remained stable over two years. Family carers with more carer burden reported poorer QOL at baseline and had poorer prognosis for QOL throughout follow-up. Depressive symptoms in carers and male carers were also associated with poorer QOL.

Similar QOL–AD scores to those observed in the present study were reported in a comparable population of 49 Norwegian co-habitant (married/unmarried) carers of persons with young-onset AD and non-AD (mean 37.9 (SD 5.5) [34].

### The trajectories of QOL–AD

We identified two groups of family carers with different trajectories in QOL–AD. The largest (better QOL) group maintained good QOL over two years with their inherent resources, network, and the services and support available to them. However, family carers in the poorer QOL-group reported significantly more burden with almost twice as high Relatives’ Stress Scale scores compared to better QOL group. Carers with greater burden at baseline had 10% increased odds of belonging to the poorer QOL-group per unit increase in the Relative Stress Scale. Carer burden has consistently been negatively associated with QOL in late-onset dementia [30]. Our findings indicate that burden also plays an important role in YOD. Early identification of burdened family carers is important as research has shown increased risk of negative health outcomes.

Caring for persons with FTD could be perceived as more stressful as behavioral symptoms are more challenging for family carers to adjust to compared to cognitive deficits [2, 8, 56]. A Dutch study on YOD reported higher burden in spouses of persons with FTD compared to AD, particularly due to higher levels of disturbing neuropsychiatric symptoms such as disinhibition and apathy [2]. Similar findings were reported in a French YOD-study [1]. In the present study, dementia subtype did not predict QOL at baseline. Families experiencing higher levels of carer burden reported poorer QOL regardless of diagnosis.

### Two-year development in QOL and associated factors among all carers assessed simultaneously

Although burden was the only predictor of QOL-group belonging, the time trend analysis identified carer burden and depressive symptoms of the carer to be negatively associated with QOL during follow-up. Family carers of persons with AD reported slightly greater but significant deterioration in QOL compared to carers of persons with FTD. Additionally, there was a significant effect of sex, both concerning the sex of the carers and the persons with dementia.

As mental health is an important component of general health and overall well-being [57], negative impact of depression on QOL could be expected. The prevalence of depression has been reported particularly high in YOD-carers, with mild to moderate depression in up to 50% of carers of persons with AD and 75–86% in FTD [13, 34]. Rosness et al. assessed QOL in carers of persons with YOD, of which 14% had FTD [34]. They found more depressive symptoms among non-AD carers compared to the AD carers using the Geriatric Depression Scale (p = .05). In contrast, one study found lower prevalence of depression in spouses of persons with FTD compared to AD, but when present, depressive symptoms were perceived as highly distressing [2].

Considering the higher prevalence of disturbing neuropsychiatric symptoms in FTD compared to AD, we were not expecting carers of persons with AD to struggle more in maintaining good QOL. However, results from another comparative study may shed some light on this controversy. A Dutch (non-YOD specific) study reported by Riedijk et al. found that despite more neuropsychiatric symptoms in persons with FTD and greater subjective burden in carers, there was no significant difference in objective measures of carer burden between carers of home-dwelling persons with FTD and AD. Carers of persons with FTD and AD with longer symptom duration had better QOL, suggesting adaptation over time. In fact, a subgroup of younger carers of persons with AD with short symptom duration reported poorer mental health on the Mental Component Summary of the Short Form 36 health survey questionnaire [3]. A prospective study of 63 dyads of persons with FTD showed stable psychological well-being and a reduction in carer burden during the two-year follow-up [58]. Perhaps the high prevalence of atypical symptoms in young-onset AD (reading/writing, agnosia, apraxia etc.) [59] may generate more practical problems for carers related to life-stage specific circumstances compared to behavioral problems in FTD. Greater awareness of progressive deterioration in functional abilities of the persons with dementia could contribute to earlier expressed needs of informal help, earlier retirement etc., and add strain on family carers. On the other hand, a Norwegian study of QOL in YOD-carers found greater awareness in persons with AD to be associated with better carer QOL [34]. In the present study, awareness did not explain the observed differences between AD- and FTD-carers.

More research is needed to identify subgroups of family carers in need of targeted QOL enhancing measures when caring for persons with young-onset AD and FTD. However, a dual pathway to improving QOL may be achieved through targeting carer burden and depression in family carers, in providing burden relief by offering practical assistance, support, psychoeducation, and the possibility of respite, and by early assessment and treatment of depression [16].

Surprisingly, we found a negative impact on QOL from being male carer. Previous studies have found associations between female dementia carers and higher levels of burden and depression, generally poorer mental and physical health, and consequently poorer QOL [60]. Gender differences and expectations inherent in the traditional sex roles are important contextual factors to the stress response in dementia care, coping, access to resources, and probably also the risk of role entrapment [61–63]. Families are generally unprepared to assume the caring role in young-onset dementia [4]. However, a possible explanation for the observed sex difference could be that men from a cultural/traditional point of view might be less capable to adapt to the premature carer role than women, as sense of self-efficacy as carer has been shown to be positively associated with QOL [64].

We lost significantly more men with YOD to follow-up compared to women, and with them also their family carers. As there was a high proportion of spousal relationships, one could assume that these dropouts represented a greater proportion of burdened females, biasing the results in favor of positive outcome. However, the distribution of female to male family carers was close to 60:40% throughout the study, maintaining a stable sex representation in our sample.

There was a significant interaction in our multiple AIC-reduced model between diagnosis and the sex of the person with dementia, as family carers of persons with AD reported better QOL when caring for women compared to men. Perhaps the higher prevalence of atypical symptoms in young-onset AD affect men and women differently regarding their functional capacities and roles within the dyad. A large recent meta-analysis showed that although certain characteristics of the persons with dementia may prove particularly stressful to the families, sex has not been identified one such factor [65].

### Strengths and limitations

The inclusion of interaction terms with time and diagnosis resulted in large number of variables for a limited number of observations but allowed us to identify significant difference in QOL between family carers based on dementia diagnosis. Limitations in sample size and/or duration of follow-up could have contributed to the non-significant result in time development of QOL.

An important limitation was including a mixed population of spouses and other carers. Characteristic differences between spousal and other types of informal carers may have influenced the outcome. The QOL–AD questionnaire used to assess QOL of the family carers was originally designed to measure QOL in people with dementia. Although it has not been validated for use in family carers it has been used in several previously studies [25, 30, 34, 66, 67] and we believe the results provided reliable and important knowledge about their QOL in a longitudinal perspective. The use of proxy reports for assessment of QOL of the persons with dementia introduces carer biases.

## Conclusions

As the family is the major provider of informal care in YOD, the physical and mental health of family carers is vital to the quality of care they provide. Family carers of persons with AD may experience greater challenges in maintaining good QOL compared to carers of persons with FTD. Multidisciplinary psychosocial interventions to reduce the stress of long-term domiciliary care, particularly focusing on burden and depressive symptoms in carers, and male carers assuming a premature carer role, may not only improve the QOL of the family carers but also benefit the persons with dementia.
